# The Effect of Test Timing on the Probability of Positive SARS-CoV-2 Swab Test Results: Mixed Model Approach

**DOI:** 10.2196/27189

**Published:** 2021-06-03

**Authors:** Roberto Benoni, Silvia Panunzi, Irene Campagna, Francesca Moretti, Giuliana Lo Cascio, Gianluca Spiteri, Stefano Porru, Stefano Tardivo

**Affiliations:** 1 Postgraduate School of Hygiene and Preventive Medicine University of Verona Verona Italy; 2 Department of Diagnostics and Public Health University of Verona Verona Italy; 3 Department of Pathology University Hospital of Verona Verona Italy

**Keywords:** close contact, COVID-19, health care workers, health surveillance, swab test timing

## Abstract

**Background:**

During the COVID-19 pandemic, swab tests proved to be effective in containing the infection and served as a means for early diagnosis and contact tracing. However, little evidence exists regarding the correct timing for the execution of the swab test, especially for asymptomatic individuals and health care workers.

**Objective:**

The objective of this study was to analyze changes in the positive findings over time in individual SARS-CoV-2 swab tests during a health surveillance program.

**Methods:**

The study was conducted with 2071 health care workers at the University Hospital of Verona, with a known date of close contact with a patient with COVID-19, between February 29 and April 17, 2020. The health care workers underwent a health surveillance program with repeated swab tests to track their virological status. A generalized additive mixed model was used to investigate how the probability of a positive test result changes over time since the last known date of close contact, in an overall sample of individuals who tested positive for COVID-19 and in a subset of individuals with an initial negative swab test finding before being proven positive, to assess different surveillance time intervals.

**Results:**

Among the 2071 health care workers in this study, 191 (9.2%) tested positive for COVID-19, and 103 (54%) were asymptomatic with no differences based on sex or age. Among 49 (25.7%) cases, the initial swab test yielded negative findings after close contact with a patient with COVID-19. Sex, age, symptoms, and the time of sampling were not different between individuals with an initial negative swab test finding and those who initially tested positive after close contact. In the overall sample, the estimated probability of testing positive was 0.74 on day 1 after close contact, which increased to 0.77 between days 5 and 8. In the 3 different scenarios for scheduled repeated testing intervals (3, 5, and 7 days) in the subgroup of individuals with an initially negative swab test finding, the probability peaked on the sixth, ninth and tenth, and 13th and 14th days, respectively.

**Conclusions:**

Swab tests can initially yield false-negative outcomes. The probability of testing positive increases from day 1, peaking between days 5 and 8 after close contact with a patient with COVID-19. Early testing, especially in this final time window, is recommended together with a health surveillance program scheduled in close intervals.

## Introduction

COVID-19, caused by SARS-CoV-2 infection, manifests as an acute respiratory distress syndrome, which requires intensive care and can lead to difficulties in the management of cases and limited hospital beds. The COVID-19 pandemic has become a challenge for health care systems because of the large in-hospital diffusion of the pathogen [1,2]. The spread of the infection may involve patients sharing the same hospital ward (ward-based contact) but can also result from infectious health care workers (12%-29% of cases) [3,4].

The ongoing COVID-19 pandemic is placing a large strain on global health care, social, and economic systems. On April 7, 2021, John Hopkins University reported a total of 132,469,663 cases and 2,874,372 deaths worldwide [5].

Italy is one of the most affected countries with 3,686,707 registered cases (as of April 7, 2021), 5% of which were health care workers [5,6]. Asymptomatic cases play an important role in the nosocomial transmission of the disease [7]; they are estimated to vary from 5% to 80% of the total number of cases [8]. Personal protective equipment (PPE) such as universal face masks and preventive actions such as SARS-CoV-2 screening programs for hospitalized patients and workers have been introduced to limit the spread of the infection [4].

Contact tracing and active surveillance implemented through SARS-CoV-2 swab tests play an important role in the containment of the disease. Very few studies have investigated the “best time to test,” particularly in situations where a close contact was detected and most of them focused on symptomatic cases only.

This study aimed to estimate how the individual probability to test positive changes over time, from the date of the last known close contact until the end of individual follow-up evaluation, and to evaluate how different scheduled surveillance time intervals might impact disease prevention.

## Methods

### Population and Setting

The University Hospital of Verona (UHV) is a high-level facility that serves an area with 922,000 inhabitants and patients from other Italian regions. During the COVID-19 pandemic, it was one of the hub centers for the Veneto region.

According to the guidelines of the Italian Ministry of Health [9], the UHV established a taskforce [10] and conducted a health care surveillance program (HSP) to ensure the safety and well-being of patients and employees and the continuity of care.

All the employees of the UHV, the staff temporarily operating at UHV structures (contractors, PhD students, and internship holders) and the University of Verona staff operating at UHV facilities were involved in the HSP and were considered health care workers (HW) for the purpose of this study. Individuals involved in the HSP with an identifiable date of close contact with a patient with COVID-19 were included in the study. The definitions of close contact are presented in Textbox 1. The study period was approximately 2 months: February 29, 2020 (data on the first swab in our database), to April 17, 2020 (set date of data collection).

**Definition of close contact with a confirmed COVID-19 case according to the Veneto region (Italy) guidelines dated March 13, 2020 [11].**

- A person cohabiting with a patient with COVID-19.
- A person who had direct physical contact with a patient with COVID-19.
- A person who had unprotected direct contact with the secretions of a patient with COVID-19.
- A person who had direct contact (face to face) with a patient with COVID-19 at a <2-meter distance, which lasted longer than 15 minutes.
- A person who has been in a closed environment (classroom, meeting room, or hospital waiting room) with a patient with COVID-19 for at least 15 minutes at a <2-meter distance.
- A health care professional or another person who provided direct assistance to a patient with COVID-19 or laboratory staff involved in handling samples of a patient with COVID-19 without the use of recommended personal protective equipment or using unsuitable personal protective equipment.

### HSP

The HSP had 2 distinct pathways for symptomatic and asymptomatic HWs who had close contact with a COVID-19–positive individual. Symptomatic individuals were tested and quarantined at home until symptom resolution. For asymptomatic individuals, an oronasopharyngeal swab sample was collected as soon as possible and successively on days 7 and 14 from the date of contact, to ascertain the negative status of the HW. Specimens were collected, in accordance with national and international recommendations [12,13], from both the oropharynx and nasopharynx by trained physicians with assistance from a professional nurse. If an individual tested positive on the swab test, 14 days of fiduciary home isolation was recommended. After this period, 2 swabs were collected consecutively within 24 h. If both tests yielded negative findings, the HWs were considered to have recovered and were allowed to return to work.

### Data Collection

At every sampling session, a short epidemiological questionnaire was administered to the HWs to collect the following data: presence of symptoms, nature of the contact (whether at the workplace or outside), age, ward working in, and personal contact details.

The AllplexTM2019-nCoV assay (Seegene Inc) was used to test the respiratory specimens. The virus was identified through multiplex real-time reverse transcriptase–polymerase chain reaction (RT–PCR) testing, which targeted 3 viral genes (E, RdRP, and N), with a detection limit of 4.8 copies/mL. The results were validated by the National Reference Laboratory of the National Health Institute [14]. Automated RNA extraction and RT–PCR were performed using Seegene NIMBUS (Seegene Inc). RT–PCR was performed using the CFX96TMDx platform (Bio-Rad Laboratories Inc) and subsequently interpreted using Seegene Viewer (Seegene Inc). Samples were considered positive at a cycle threshold (Ct) value of ≤40 for at least 1 of the 3 target genes. Microbiology laboratory data (swab results and dates) and data from the questionnaires were then merged into a unique database.

### Ethics Statement

According to Decree-Law N.14 of March 9, 2020, personal data can be collected to guarantee public health and ensure the diagnosis and assistance of the infected individuals in the context of the COVID-19 emergency [15]. All data were collected exclusively for the purpose of the health surveillance program and were anonymized and presented in an aggregated format to ensure the privacy of the participants. The study conformed to the ethical standards of the 1964 Declaration of Helsinki and its later amendments.

### Statistical Analysis

A data exploratory analysis was first conducted to investigate the characteristics of the HWs. Categorical data were compared using the chi-square test, and the Mann-Whitney (2-sample Wilcoxon) test was performed for continuous variables and to compare time-to-test positivity between groups of individuals (no censoring was carried out in this analysis; therefore, survival techniques could be discharged).

Generalized additive mixed models (GAMM) for binomially distributed data were used to investigate how the probability of a positive test result changes over time since the last known close contact date declared by the HW [16]. A continuous smooth function was used to model the effect of the number of days since close contact on the probability of testing positive and individual-specific random intercepts were included in the model to account for intraclass correlation of observations from among the same participants.

The same analysis was also performed to assess the probability of testing positive in the subset of individuals who eventually tested positive but initially presented a negative swab test finding. In addition to the aforementioned principal analyses, alternative surveillance time intervals were assumed to investigate how they might influence the results of our analysis. Specifically, for each individual, swabs following the first one, taken as soon as possible, were simulated at closer intervals (3 and 5 days) than the standard 7-day interval planned by the HSP. The length of surveillance between a testing date and the next one was “virtually” modified with a mathematical shift as shown below.

An index to enumerate individual swabs was generated as follows:


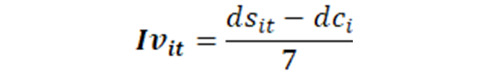


where *ds* and *dc* are the dates of the individual swab tests and of close contact, respectively, *i* identifies each of the individuals included in the study, and *t* is the index identifying each individual’s test finding.

In our HSP, the fixed surveillance interval between swabs was 7 days. The *Iv* for the first swab (*Iv_i1_*) was assumed to be 0 because it was considered unmodifiable by HSP timing.

Thereafter, we formulated a GAMM model introducing the index time as follows:

Y_i_ ~ Bin (n_i_, π_i_); π_i_ = probability of a positive test

Logit(π_it_) = β_i_[f{(ds_i1_ – dc_i_) + (Iv_it_ × It)}] + b_i0_ + ε_it_

where *ds_1_* and *dc* are the date of the first swab and the date of close contact, respectively, *i* is the individual index, and *t* is the time observation index. *It* and *Iv* are the fixed surveillance interval time and the index visit, previously exposed, and *f {(ds_i1_ – dc_i_) + (Iv_it_ × It)}* indicates a smooth function with penalized splines; furthermore, *b_i0_* is the individual-specific random intercept in the model.

A *P* value of <.05 was considered signiﬁcant. All analyses were performed using R software (version 3.5.2, The R Foundation).

## Results

During the study period, 2071 of 6092 (34%) HWs currently employed had close contact with a confirmed patient with COVID-19. Among them, 191 (9.2%) yielded positive findings on oropharyngeal swab tests. This proportion was not significantly different for the sex (*P*=.25) and age (*P*=.31) of the individuals included in this study (Table 1). Among the COVID-19–positive individuals, 88 (46%) presented at least mild symptoms (such as cough, fever, dyspnea, sore throat, anosmia, and ageusia). The median time of symptom onset after close contact was 4.0 (IQR 1.0-6.3) days and was not significantly different for sex (*P*=.75) and age (*P*=.48).

In 25.7% of the individuals who tested positive (n=49), the first swab test after close contact yielded a negative result (Table 1). The occurrence of an initial negative swab test result was not associated with sex, age, or symptoms. In 9 of these individuals (4.7% of all infected HWs), the second swab test yielded a negative finding. The median time between close contact and the first swab test was 3 days for the group with an initial negative finding, whereas it was 4 days for those who tested positive on the first swab test (*P*=.06).

**Table 1:** Characteristics of health care workers at University Hospital of Verona (Italy) distinguished by SARS-CoV-2 swab test results and the presence of symptoms (data collected between February 29 and April 17, 2020).

Characteristics		Infected health care workers with an initial negative swab test finding after close contact with a patient with COVID-19				Positive swab test finding among health care workers after close contact with a patient with COVID-19			Symptomatic health care workers with COVID-19		
		Yes (n=49)	No (n=142)		*P* value^a^	Yes (n=191)	No (n=1880)	*P* value	Yes (n=88)	No (n=103)	*P* value
**Sex, n (%)**					.40			.25			.20
	Male	15 (31)		55 (39)		70 (37)	607 (32)		37 (42)	33 (32)	
	Female	34 (69)		87 (61)		121 (63)	1273 (68)		51 (58)	70 (68)	
Age (years), median (IQR)		40.9 (30.0-52.4)		47.9 (32.9-55.3)	.06	46.7 (31.8-54.8)	45.1 (32.2-53.2)	.31	48.9 (36.9-54.8)	41.5 (30.3-54.7)	.08
**Symptoms, n (%)**					.31	N/A^b^	N/A	N/A	N/A	N/A	N/A
	Yes	19 (39)		69 (49)							
	No	30 (61)		73 (51)							
Days from close contact to initial swab sample collection, median (IQR)		3.0 (1.0-6.0)		4.0 (2.0-8.0)	.06	N/A	N/A	N/A	N/A	N/A	N/A

^a^*P* values were computed using the chi-square test and the nonparametric Mann–Whitney *U* test.

^b^N/A: not applicable.

Since all HWs were checked over time with repeated swabs under the HSP, it was possible to calculate the proportion of false-negative findings from among the total number of individuals who tested negative (false omission rate). The false omission rate was 2.5%.

The median time from the date of individual close contact to the first positive finding on the swab test was estimated. In the overall sample of HWs, this median time was 7 (IQR 4-11) days. Considered separately, the median time for HWs who tested negative on their initial swab test and that for HWs who did not was 10 days and 4 days, respectively. No significant differences were observed in the time to the first positive finding on the swab test with respect to sex (*P*=.62), age (*P*=.47), or clinical manifestations (*P*=.39).

**Figure 1:**
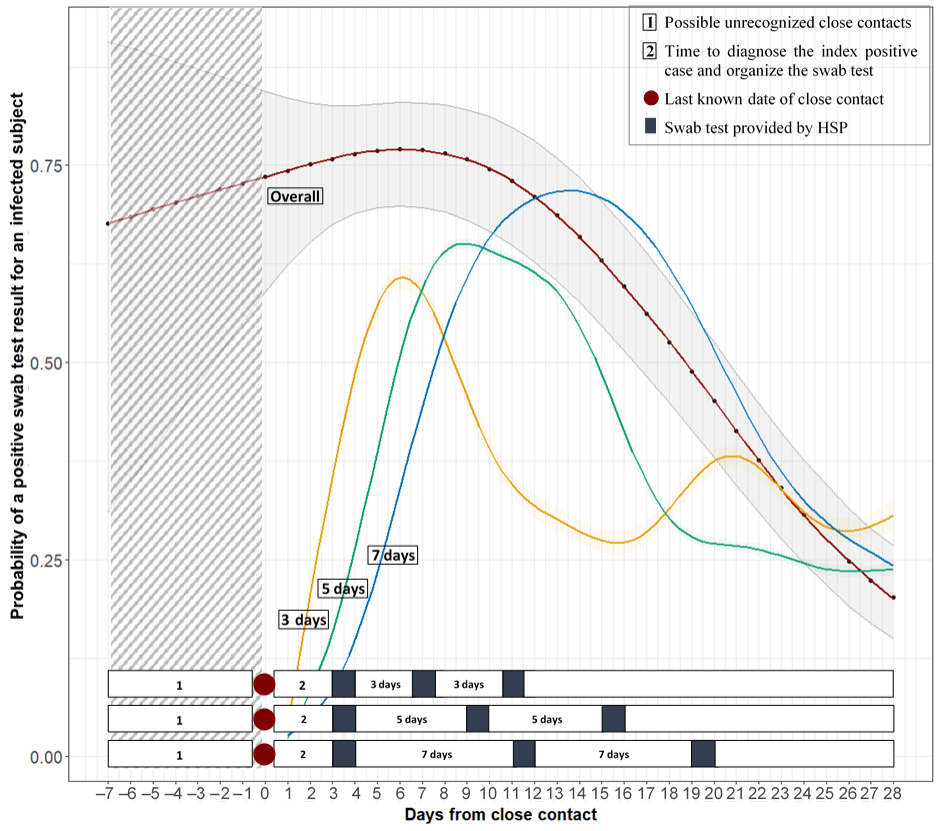
Graphical predictions from the generalized additive mixed models. The 4 curves represent the predicted probabilities of having a positive test related to the number of days since the last known date of close contact among all health care workers infected with SARS-CoV-2 (overall curve) and in the subgroups of those infected with SARS-CoV-2 with an initial negative swab test result in the 7-day standard surveillance interval and simulating 2 different time intervals of 3 and 5 days. The bars show the time course of the health surveillance program in the 3 different time intervals related to the probability curves. HSP: health surveillance program.

In the GAMM model that considered the whole group of COVID-19–positive individuals, the probability of a positive swab test result increased from 0.74 on day 1 to 0.77 on day 5 after close contact (Table 2). This probability peaked between the fifth and eighth days (0.77) and then decreased during subsequent days since close contact (Figure 1). All the predicted model probabilities for the first to the 21st day from close contact are indicated in Table 2.

**Table 2:** Predicted probabilities of the generalized additive mixed models for a positive swab test result related to the number of days since close contact in the whole group of infected health care workers at the University Hospital of Verona (model was fitted with data collected between February 29 and April 17, 2020).

Day	π (0.95% CI)
1	0.74 (0.62-0.84)
2	075 (0.65-0.83)
3	0.76 (0.67-0.83)
4	0.76 (0.69-0.83)
5	*0.77 (0.69-0.83)* ^a^
6	*0.77 (0.69-0.83)*
7	*0.77 (0.69-0.83)*
8	*0.77 (0.69-0.83)*
9	0.76 (0.68-0.82)
10	0.75 (0.67-0.81)
11	0.73 (0.65-0.79)
12	0.71 (0.63-0.78)
13	0.69 (0.60-0.76)
14	0.66 (0.58-0.73)
15	0.63 (0.55-0.71)
16	0.60 (0.51-0.67)
17	0.56 (0.48-0.64)
18	0.53 (0.45-0.60)
19	0.49 (0.41-0.56)
20	0.45 (0.38-0.53)
21	0.41 (0.34-0.49)

^a^Italicized values indicate the days where the probability of testing positive for COVID-19 on the swab test peaks.

The same model was used to analyze the change over time of the probability of a positive swab test finding in the subset of HWs with an initial negative swab test result in the standard HSP 7-day interval and in the alternative “simulated schedule time” for surveillance intervals of 3 days and 5 days (Table 3).

In the 3 intervals (3, 5, and 7 days), the highest peak was observed on the sixth day, between the ninth and tenth days, and between the 13th and 14th days, respectively, with a probability of a positive swab test result of 0.61, 0.65, and 0.72, respectively (Figure 1). This probability then decreased with time in all surveillance interval models except for the 3 day interval, in which a secondary tail peak was obtained on the 21st day with a probability of 0.38. All the predicted model probabilities for the 3 assumed surveillance intervals and for days 1 to 28 from close contact are indicated in Table 3.

**Table 3:** Predicted probabilities of the generalized additive mixed model for a positive swab test result for COVID-19 in relation with the number of days since close contact with a patient with COVID-19, among infected health care workers from University Hospital of Verona (Italy) with an initial negative swab test result (the model was fitted with data collected between February 29 and April 17, 2020). Three different scenarios were included for the surveillance time interval (3, 5, and 7 days).

Day since close contact with a patient with COVID-19	3 days, π (0.95% CI)	5 days, π (0.95% CI)	7 days, π (0.95% CI)
1	0.07 (0.02-0.23)	0.03 (0.01-0.16)	0.03 (0.00-0.14)
2	0.17 (0.07-0.35)	0.07 (0.02-0.23)	0.05 (0.01-0.18)
3	0.34 (0.20-0.51)	0.15 (0.06-0.32)	0.09 (0.03-0.24)
4	0.50 (0.35-0.65)	0.26 (0.14-0.44)	0.15 (0.06-0.31)
5	*0.59 (0.45-0.72)* ^a^	0.40 (0.25-0.56)	0.23 (0.12-0.41)
6	*0.61 (0.46-0.75)*	0.51 (0.37-0.66)	0.34 (0.20-0.51)
7	*0.58 (0.42-0.72)*	0.59 (0.45-0.72)	0.44 (0.30-0.60)
8	0.52 (0.36-0.67)	0.63 (0.48-0.76)	0.54 (0.39-0.67)
9	0.45 (0.31-0.61)	*0.65 (0.49-0.78)*	0.61 (0.47-0.73)
10	0.40 (0.27-0.54)	*0.65 (0.47-0.79)*	0.66 (0.51-0.78)
11	0.35 (0.24-0.49)	0.64 (0.45-0.79)	0.69 (0.54-0.81)
12	0.32 (0.20-0.46)	0.62 (0.43-0.77)	0.71 (0.55-0.83)
13	0.30 (0.18-0.45)	0.59 (0.40-0.75)	*0.72 (0.54-0.84)*
14	0.28 (0.16-0.45)	0.54 (0.37-0.71)	*0.72 (0.53-0.85)*
15	0.27 (0.14-0.46)	0.48 (0.33-0.64)	0.71 (0.52-0.85)
16	0.27 (0.14-0.47)	0.41 (0.28-0.57)	0.69 (0.49-0.84)
17	0.29 (0.14-0.50)	0.35 (0.23-0.50)	0.66 (0.46-0.82)
18	0.32 (0.15-0.55)	0.30 (0.19-0.45)	0.62 (0.43-0.78)
19	0.35 (0.15-0.61)	0.28 (0.16-0.43)	0.57 (0.39-0.74)
20	0.37 (0.16-0.65)	0.27 (0.15-0.43)	0.52 (0.35-0.68)
21	*0.38 (0.15-0.69)*	0.26 (0.14-0.44)	0.46 (0.31-0.62)
22	0.37 (0.12-0.71)	0.26 (0.14-0.45)	0.41 (0.27-0.56)
23	0.34 (0.10-0.72)	0.26 (0.13-0.45)	0.36 (0.24-0.51)
24	0.31 (0.07-0.73)	0.25 (0.12-0.45)	0.33 (0.21-0.48)
25	0.29 (0.06-0.74)	0.24 (0.10-0.46)	0.30 (0.18-0.45)
26	0.28 (0.05-0.75)	0.23 (0.09-0.47)	0.28 (0.16-0.43)
27	0.29 (0.04-0.78)	0.23 (0.08-0.50)	0.26 (0.14-0.42)
28	0.31 (0.04-0.82)	0.24 (0.08-0.54)	0.24 (0.13-0.41)

^a^Italicized values indicate the days where the probability of testing positive for COVID-19 on the swab test peaks.

## Discussion

### Principal Findings

Identification and tracking of close contacts are a fundamental strategy to prevent the spread of COVID-19. During the study period, approximately one-third of UHV HWs claimed that they had close contact with a patient with COVID-19. Among them, 9.2% tested positive on the oronasopharyngeal swab test. Other studies have reported similar results among HWs with regard to both the occurrence of close contacts and the prevalence of positive cases [17,18]. Nosocomial transmission of SARS-CoV-2 with outbreaks occurring in different wards has been reported in several countries [19,20].

In our study sample, approximately half of the HWs who tested positive were asymptomatic. The consistency of this finding with those of previous studies [21,22] may justify the need to test individuals regardless of clinical manifestation, especially in a health care setting, to avoid disease spread from asymptomatic cases [23].

The median time for the occurrence of symptoms, starting from the date of declared close contact, for symptomatic individuals was estimated to be 4 days. The incubation time of SARS-CoV-2 is still being debated: a meta-analysis conducted mostly on Chinese data estimated a mean incubation time of approximately 5 days [24], while the European Centre for Disease Prevention and Control reports a median incubation period between 5 and 6 days, ranging from 2 to 14 days [25].

RT–PCR analysis of oronasopharyngeal swab samples has been recognized as the most reliable test to identify and ascertain SARS-CoV-2 infection; however, the sensitivity and specificity of the swab test are still being discussed [26,27]. Two reviews reported sensitivities ranging between 63% and 98% [28,29]. The specificity of the test was reported to be higher (95%) [27].

In this study, 58 swab tests yielded negative findings before a positive finding was obtained in the subsequent test. The false-negative rate (the test does not initially detect the infection when one actually acquires it) in our sample was 22.3%. This result may have different explanations apart from the intrinsic sensitivity of the RT–PCR test. First, the rate of false-negative results may change in accordance with how long the infection has been acquired. The time between the date of the close contact and the date of the first specimen collection was not different between the 2 groups of positive and negative initial swabs, with comparable demographic characteristics, thus limiting the bias of an incorrect sampling timing (Table 1). However, some individuals may have presented a longer incubation period before the virus could be detected in a test, thus leading to false-negative results despite the infection already having been acquired. False-negative results are influenced even by the sampling quality of the swab. In particular, when the viral load is low, the specimen collection technique could be a source of diagnostic errors [30]. It is also worth noting that the RT–PCR assays used herein had a Ct of 40. The Ct is a semiquantitative value. A lower Ct value corresponds to a higher quantity of viral genetic material in the sample, which can be considered an approximation of the viral load. However, viruses have been isolated from laboratory cell cultures from samples exhibiting Ct values above 36 [31]. It may be possible that some of the samples, considered negative because of their high Ct, still had a very low quantity of viral genetic material, which increased in the subsequent days. In the study sample, the estimated probability of incorrectly ruling out a case of COVID-19 on the basis of a false-negative test was 2.5%. Long et al [32] reported similar results upon assessing 678 patients who underwent repeated testing. It must be considered that this probability is influenced by not only test sensitivity but also disease prevalence.

Starting from the assumption that false-negative results may be obtained, the median time to a positive swab test result was measured in the whole group and then separately, distinguishing between HWs with an initially negative finding and those with an initially positive finding on the swab test. The estimated median time from close contact to the first positive test was 7 days overall, 4 days in the group of HWs who presented an initial positive result, and 10 days for those who presented an initial negative finding on the swab test. This can be partly attributed to the HSP that planned a swab every 7 days for individuals with close contact. The surveillance time interval plays a crucial role in the detection of COVID-19–positive individuals and influences the spread of the infection.

As a repeated testing strategy is considered important to overcome false-negative results, especially in a health care setting [33,34], a GAMM model was applied to investigate how the probability of obtaining a positive swab test result may change over time from the last known date of close contact of that individual and ultimately to discern a possible “best time to test” after a close contact.

In our sample, the probability of a positive swab was 0.74 on day 1 and peaked (0.77) between days 5 and 8 after close contact. This high probability retrieved from the first day after close contact can be explained by the fact that the contact causing infection may have occurred on a previous date than the last one that was recognized and reported by the HW (Figure 1). Individuals who test positive have shown to be infective since the preclinical and subclinical stages of the disease [24,35,36], and it was reported that the replication rate of SARS-CoV-2 peaks 2 days post infection, with an increase in the virus titer detectable already in the first 24 h post inoculation [37]. Two studies conducted only on symptomatic individuals reported the maximum probability of a positive swab test result in the initial days following the onset of symptoms [34,38].

In our sample, 25.7% and 4.7% of infected HWs required 2 and 3 tests, respectively, to be confirmed as positive, consistent with a previous report [39]. Considering this group, the probability peak (0.72) was obtained approximately 13-14 days after close contact for the 7-day standard HSP surveillance interval.

When simulating a 3- and 5-day interval model, the probability of obtaining a positive swab test result peaked on the fifth to seventh and the ninth to tenth days, respectively. In the 3-day interval model, the time of the highest probability was similar to that estimated for the whole group of individuals who tested positive (Figure 1). The function for the 3-day interval shows 2 peaks, one between days 5-7 and another on day 21 after close contact. For screening intervals of 5 and 7 days, the function seems to include the 2 sets of swabs obtained on the same individual to ascertain positivity first and negativity thereafter, under a unique curve interval because of the wider intervals. Simulations of the screening time intervals narrower than the 7-day standard HSP surveillance interval have shown that the probability of testing positive can be detected earlier.

### Limitations

One of the limitations of this study is that HWs have self-reported the dates of close contact, which may have led to recall bias errors. On the other hand, the HSP, by continuously monitoring HWs over time, permitted the registration of the updated last dates of close contact and to identify true-negative cases. HWs who tested positive at least once were considered true-positive cases, consistent with a previous report [38], even if the specificity of the swab test may have influenced the results.

Contact tracing has been one of the main strategies to keep the pandemic under control; however, only a few studies have investigated the relationship between close contact and a positive swab test result. Monitoring of HWs through swab testing in the hospital setting is particularly important because it may prevent large-scale disease spread.

### Conclusions

This study is one of the first to explore the best scheduling time in an HSP for HWs. A call for algorithms has been made to detail the exact pathway to monitor the safety of HWs [40]. In a hospital setting, several unrecognized close contacts that cause infection may occur. Having shown that the probability of a positive swab test result for COVID-19 is already high in the first days and peaks between the fifth and eighth days after the last known close contact, early testing, especially within this time window would be advisable. Narrowing of surveillance intervals between swabs in the first 10 days might be recommended, since negative results may be obtained initially. Many European countries are currently facing a third wave of COVID-19. Ensuring the safety of patients and HWs and the continuity of care are fundamental in this situation. The correct use of the appropriate PPE is pivotal in preventing new infections; however, when an accidental close contact with a positive case occurs, it is mandatory to monitor HWs through swab testing. Many positive individuals may be asymptomatic or pauci-symptomatic; therefore, the recommendation is to test all HWs regardless of their clinical manifestations.

## Abbreviations

Ct: cycle threshold
GAMM: generalized additive mixed model
HSP: health surveillance program
HW: health care worker
PPE: personal protective equipment
RT–PCR: reverse transcriptase–polymerase chain reaction
UHV: University Hospital of Verona
